# Metabolic syndrome detection with biomarkers in childhood cancer survivors

**DOI:** 10.1530/EC-20-0144

**Published:** 2020-06-18

**Authors:** V G Pluimakers, M van Waas, C W N Looman, M P de Maat, R de Jonge, P Delhanty, M Huisman, F U S Mattace-Raso, M M van den Heuvel-Eibrink, S J C M M Neggers

**Affiliations:** 1 Princess Máxima Centre for Paediatric Oncology, Utrecht, The Netherlands; 2 Department of Paediatric Oncology/Haematology, Erasmus MC–Sophia Children’s Hospital, Rotterdam, The Netherlands; 3 Department of Public Health, Erasmus MC, Rotterdam, The Netherlands; 4 Department of Haematology, Erasmus MC, Rotterdam, The Netherlands; 5 Department of Clinical Chemistry, Erasmus MC, Rotterdam, The Netherlands; 6 Section Endocrinology, Department of Medicine, Erasmus MC, Rotterdam, The Netherlands; 7 Section Geriatric Medicine, Department of Medicine, Erasmus MC, Rotterdam, The Netherlands

**Keywords:** childhood cancer survivor, metabolic syndrome, nephroblastoma, neuroblastoma, principal component analysis, biomarker

## Abstract

**Purpose:**

Augmented survival of childhood nephroblastoma and neuroblastoma has increased long-term side effects such as metabolic syndrome (MetS). Risk stratification is difficult after abdominal radiation because waist circumference underestimates adiposity. We aimed to develop a strategy for determining MetS in irradiated survivors using an integrated biomarker profile and vascular ultrasonography.

**Methods:**

The NCEP-ATPIII MetS-components, 14 additional serum biomarkers and 9 vascular measurements were assessed in a single-centre cohort of childhood nephroblastoma (*n* = 67) and neuroblastoma (*n* = 36) survivors and controls (*n* = 61). Multivariable regression models were used to study treatment effects. Principal component analysis (PCA) was used to study all biomarkers in a combined analysis, to identify patterns and correlations.

**Results:**

After 27.5 years of follow-up, MetS occurred more often in survivors (14%) than controls (3%). Abdominal radiotherapy and nephrectomy, to a lesser extent, were associated with MetS and separate components and with several biomarker abnormalities. PCA of biomarkers revealed a pattern on PC1 from favourable lipid markers (HDL-cholesterol, adiponectin) towards unfavourable markers (triglycerides, LDL-cholesterol, apoB, uric acid). Abdominal radiotherapy was associated with the unfavourable biomarker profile (β = 1.45, *P* = 0.001). Vascular measurements were not of added diagnostic value.

**Conclusions:**

Long-term childhood nephro- and neuroblastoma survivors frequently develop MetS. Additional assessment of biomarkers identified in PCA – adiponectin, LDL, apoB, and uric acid – may be used especially in abdominally irradiated survivors, to classify MetS as alternative for waist circumference. Vascular ultrasonography was not of added value.

## Introduction

Over the past decades, survival rates of childhood nephroblastoma and neuroblastoma have increased to respectively ~90% and ~40–95% (strongly dependent on stadium) (1, 2). These tumours are of embryonic origin, with a peak incidence under the age of 5 years and a presentation predominantly in the abdomen. Treatment often consists of a combination of surgery (nephrectomy and/or adrenalectomy), radiotherapy and/or intensive chemotherapy. Because of increased survival rates, long-term side effects, such as adiposity, insulin resistance, dyslipidaemia and hypertension, have become more prominent, particularly after treatment with abdominal radiotherapy (3, 4, 5). These risk factors for diabetes mellitus and cardiovascular disease interact and cluster together as metabolic syndrome (6, 7, 8).

In order to prevent the development of diabetes mellitus and cardiovascular disease, it is important to identify survivors at risk of developing (components of) MetS and to diagnose and treat them in a timely fashion (9). Risk stratification in childhood cancer survivors (CCS) with the classic criteria for MetS components can be difficult. This is due to the underestimation of adiposity by waist circumference, waist-hip ratio and BMI, in particular after abdominal radiotherapy has been applied (3, 10). Also, because CCS are relatively young, absolute occurrence rates of cardio- and cerebrovascular events are low, even though they are at higher relative risk (11, 12, 13, 14).

There is evidence that measurement of (visceral) fat by DXA-scan is a better indicator of adiposity and, hence, a better predictor for cardiovascular disease (3, 10), but this is a costly and time consuming test. Therefore, in addition to the serum biomarkers triglycerides and HDL cholesterol that are already included in the definition of MetS, several other biomarkers have been suggested as surrogate markers for development of MetS and cardiovascular disease. These additional biomarkers include low-density lipoprotein (LDL) cholesterol, adiponectin, uric acid, C-reactive protein and cystatin C (15, 16, 17). Also, it has been proposed that metabolic biomarkers are more clinically useful for risk prediction of diabetes and cardiovascular disease when analysed as a combination reflecting different pathophysiologic pathways, to reveal underlying patterns or clusters of dysmetabolic development (18). In addition, vascular ultrasound measurements, such as carotid intima media thickness (CIMT), pulse pressure amplification (PPA) and pulse wave velocity (PWV), have also been proposed as surrogate markers for cardiovascular disease (19, 20). So far, no studies have reported on the value of these additional biomarkers and vascular ultrasound measurements in CCS.

The aim of this cross-sectional study was to develop a strategy for determining MetS even in abdominally irradiated long-term survivors of childhood nephro- and neuroblastoma, using an integrated biomarker profile, based on principal component analysis and vascular ultrasound measurements.

## Patients and methods

### Patients

Patients were actively recruited as described before (3). Briefly, all long-term (5 or more years after treatment) adult survivors of childhood nephro- and neuroblastoma (except for survivors of neuroblastoma stage 4s who did not receive surgery, radiotherapy or chemotherapy), treated between 1961 and 2004 in the Erasmus MC/Sophia Children’s Hospital, Rotterdam, The Netherlands, that visited the late effects outpatient clinic regularly were invited to participate in this cross-sectional study. The study was approved by the MREC Erasmus MC Rotterdam (trial NL2685, study period 2009–2012). Survivors were asked to invite potential control subjects such as siblings, friends or neighbours, preferably of the same sex and within an age range of 5 years. Written informed consent was obtained from all participants.

### Data collection

Disease and treatment data were obtained from the medical records. Detailed data regarding surgery were confirmed from the original surgical and pathological reports. Information on medication use (statins, antidiabetic, antihypertensive), smoking and socio-economic status was collected using a self-designed questionnaire. Weight was measured with underwear only to the nearest 0.1 kg with a standard clinical balance. Height was measured to the nearest millimetre using a Harpenden Stadiometer. BMI was calculated (weight(kg)/height(cm)^2^). Waist circumference was measured between lower rib and iliac crest to the nearest centimetre. Blood pressure was measured with the subject in sitting position after an hour of rest on the right arm with the Dinamap® Procare and was defined as the mean of three measurements. Components of MetS were defined using the NCEP-ATPIII classification: waist ≥102(men)/88(women) cm, triglycerides ≥1.7 mmol/L or use of statins, HDL cholesterol ≤1.03(men)/1.29(women) mmol/L or use of statins, blood pressure ≥130/≥85 mmHg or use of antihypertensives, fasting glucose ≥5.6 mmol/L or antidiabetic treatment, with three or more criteria required for the diagnosis MetS (6). The occurrence of MetS and components in the current study population have been previously published (3).

### Laboratory measurements

Fasting venous blood samples were taken before 10:00 h. In addition to the biomarkers in the NCEP-ATPIII classification, 13 biomarkers were assessed: free fatty acids (FFA), apolipoprotein(apo)-A1, apoB, LDL cholesterol (measured, not calculated), leptin, adiponectin, lipoprotein(a) (Lp(a)), insulin, cystatin C, uric acid, urea, creatinine and hsCRP. Homeostasis model assessment (HOMA) was used as an estimate of insulin resistance and beta-cell function calculated from glucose and insulin concentrations (21). Also, antithrombin, protein C, protein S, diluted Russell’s viper venom time and von Willebrandfactor antigen were measured to exclude subjects with possible non-cancer-therapy-related coagulation problems (results not reported as outcome variables).

### Vascular ultrasound measurements

Central systolic and diastolic blood pressure were assessed with the SphygmoCor (AtCor Medical, Sydney, Australia), which calculates aortic blood pressure from brachial pulse wave. Brachial and central pulse pressure (PP, the difference between systolic and diastolic blood pressure) were calculated, as well as pulse pressure amplification (PPA, brachial divided by central PP). Measurements of the carotid artery were performed with the subject in supine position, the head tilted slightly towards the contralateral side. After 5 min of rest, diameter of the common carotid artery (CCA), carotid intima media thickness (CIMT) and distensibility were measured with a duplex scanner (operating frequency 7.5 MHz, Pie Medical Imaging, Maastricht, The Netherlands) during six non-consecutive heartbeats and reported as mean values. Distensibility coefficient (DC) was calculated using the following formula: (((2000 × distensibility)/diameter)/PP × 133.22) (22). Pulse wave velocity (PWV) was also measured with the subject in supine position, with the Complior (Alam Medical, Saint-Quentin-Fallavier, France), which simultaneously records pulse waves at the carotid and femoral arteries (PWV = carotid-femoral distance/time delay).

### Statistical analysis

Statistical analysis was performed using R version 3.5.1 (R Foundation for Statistical Computing, Vienna, Austria). Baseline characteristics were compared between survivors and controls as well as nephroblastoma survivors compared to neuroblastoma survivors, using Fisher’s exact test and Chi-squared test for categorical variables and by bootstrapping the difference in median values for continuous variables.

Occurrence of MetS and MetS components was compared between survivors and controls using Fisher’s exact test and the Chi-squared test, respectively. Serum biomarkers and vascular ultrasound measurements were compared between survivors and controls by bootstrapping the difference in median values, for which the 95% CI was calculated with percentiles.

The effect of abdominal radiotherapy and nephrectomy on (components of) MetS, the serum biomarkers and the vascular parameters was tested with univariable logistic and bootstrap linear regression models and, when significant, also in multivariable regression models, adjusting for age, sex, smoking and socio-economic status. There was no need to adjust for use of steroids, as these are not administered in treatment protocols of these malignancies or for adrenalectomy, because this would lead to overcompensation, as we previously published (23).

The serum biomarkers were also analysed by principal component analysis (PCA), to identify correlations and discriminative patterns and to reduce the effects of multiple testing. PCA is an unsupervised, combined analysis of all biomarkers that explains most of the variance in two principal components (PC1 and PC2) and the relative contribution of each biomarker to these principal components. With this method, the individual, unbiased contribution of each biomarker is calculated. PCA was performed on the correlation matrix, which means that all variables are standardized to Z-scores. Missing values were imputed with the median (except for cystatin C, which was missing in 15% of participants and therefore was predicted with R package *mice* based on age, sex and the other kidney function variables). The effect of abdominal radiotherapy and nephrectomy on PC1 and PC2 was tested with linear regression models.

For the analyses of serum biomarkers, vascular ultrasound measurements and PCA, participants using relevant medication were excluded. A *P*-value <0.05 was considered as statistically significant.

## Results

### Study population

Eighty-eight nephroblastoma survivors were invited to participate in the study, of whom 67 (39 males) agreed (76%). Fifty-five neuroblastoma survivors were invited, of whom 36 (15 males) agreed to participate (65%). Survivors who did not participate were similar to participating survivors with respect to baseline characteristics. In total, 61 controls were included (33 males), 37 of whom were siblings and 24 were partner or friend. Baseline and treatment characteristics are depicted in Table 1. Median age was 30 and 31.8 years for survivors and controls, respectively, and median follow-up time of survivors was 27.5 years (range 6.4–48.9 years). Systolic and diastolic blood pressure were higher among survivors, whereas physical activity, smoking behaviour and socio-economic status were not significantly different between survivors and controls. Within survivors, nephroblastoma survivors were older at diagnosis and had been treated more often with nephrectomy and abdominal radiotherapy (Supplementary Table 1, see section on supplementary materials given at the end of this article). None of the study participants had experienced a cardiac event or stroke at time of inclusion in the study.

**Table 1:** Baseline characteristics of included survivors and controls.

	Survivors	Controls	Bootstrap 95% CI	*P*-value
Number	103 (67 nephro-, 36 neuroblastoma)	61		
Male sex	54 (52.4%)	33 (54.1%)	n.a.	0.96^c^
Age at follow-up (years)^a^	30.0 (25.2–37.9)	31.8 (23.3–40.0)	(−7.2;2.4)	0.33^d^
Age at diagnosis (years)^a^	2.3 (0.8–5.0)	n.a.		
Follow-up time^b^ (years)^a^	27.5 (20.1–31.6)	n.a.		
BMI (kg/m^2^)^a^	24.3 (21.3–26.3)	24.2 (22.1–27.2)	(−1.8;1.6)	0.84^d^
Systolic BP (mmHg)^a^	124 (117–133)	118 (111–126)	(0.3;10.0)	0.026^d^*
Diastolic BP (mmHg)^a^	76 (72–83)	72 (66–78)	(0.7;7.8)	0.012^d^*
Medication use		0		
Lipid-lowering	4 (3.9%)	2 (3.3%)	n.a.	0.30^e^
Diabetes	6 (5.8%)		n.a.	0.085^e^
Antihypertensive	6 (5.8%)		n.a.	0.71^e^
Physical activity score^a^	7695 (6390–10,890)	8080 (6465–12,278)	(−2947;1264)	0.71^d^
Smoking			n.a.	0.62^c^
Non-smoker	62 (60%)	32 (53%)		
Former smoker	15 (14.6%)	10 (16.4%)		
Smoker	26 (25%)	19 (31%)		
Socio-economic status			n.a.	0.31^e^
Low	22 (21.4%)	10 (16.4%)		
Medium	36 (35.0%)	29 (47.5%)		
High	45 (43.7%)	22 (36.1%)		
Nephrectomy	74 (71.8%)	n.a.		
Adrenalectomy	47 (45.6%)	n.a.		
Abdominal radiotherapy	42 (40.8%)	n.a.		
Pancreas	32 (31.1%)			
Flank	17 (17.0%)			
Cumulative dose radiotherapy (Gy)^a^	21 (20–30)	n.a.		
Chemotherapy	90 (87.4%)	n.a.		
Vincristine	65 (63.1%)			
Actinomycine	48 (46.6%)			
Anthracyclines	30 (29.1%)			
Cyclofosfamide	31 (30.1%)			
Cisplatin	7 (6.8%)			
Teniposide	6 (5.8%)			
Dacarbazine	2 (1.9%)			
Ifosfamide	2 (1.9%)			
Corticosteroids	2 (1.9%)	n.a.		

Significance codes: 0 *** 0.001 ** 0.01 * 0.05.

^a^Presented as median (IQR); ^b^Time after cessation of treatment; ^c^Chi-squared test; ^d^Bootstrapped difference in medians; ^e^Fisher’s exact test.

BP, blood pressure; n.a., not applicable.

### Classic MetS components, biomarkers and vascular ultrasound measurements, as compared to controls

MetS, as defined by the presence of at least three of the NCEP-ATPIII criteria, was present in 14 survivors (14%) and 2 controls (3%, *P* = 0.032), as previously described (3). Thirty-four survivors (33%) revealed at least two MetS criteria, compared to 12 controls (20%, *P* = 0.074). Hypertension and treatment for hypertension occurred significantly more often in survivors than controls, whereas the other MetS components did not differ significantly between groups (Table 2).

**Table 2:** Occurrence of MetS and components in survivors and controls.

	Survivors (*n* = 103)	Controls (*n* = 61)	*P*-value
Metabolic syndrome (≥3 components)	14%	3%	0.032*^a^
≥2 MetS components	33%	20%	0.074^a^
Abdominal obesity (waist circumference ≥102 (men)/88 (women) cm)	8%	11%	0.61^b^
High triglycerides (≥1.7 mmol/L) or treatment	23%	10%	0.052^b^
Low HDL cholesterol (≤1.03 (men)/1.29 (women) mmol/L) or treatment	29%	18%	0.16^b^
High blood pressure (≥130/≥85 mmHg) or treatment	35%	15%	0.007**^b^
High glucose (≥5.6 mmol/L) or treatment	22%	11%	0.20^b^

Significance codes: 0 *** 0.001 ** 0.01 * 0.05.

^a^Fisher’s exact test; ^b^Chi-squared test.

Triglycerides (∆ = 0.17 mmol/L, *P* = 0.036), cystatin C (∆ = 0.06 mg/L, *P* = 0.002) and creatinine levels (∆ = 5 mg/mmol, *P* = 0.014) were significantly higher in survivors compared to controls (Table 3). The other additional biomarkers were not different between survivors and controls. All coagulation markers were within the reference range in all participants (data not shown).

**Table 3:** Comparison of serum biomarkers and vascular parameters between survivors and controls.

Variable	Survivors (*n* = 103)^a^	Controls (*n* = 61)^a^	95% CI^b^	*P*-value^b^
Biomarkers				
Lipid metabolism^c^				
Triglycerides (mmol/L)	0.96 (0.72–1.41)	0.79 (0.63–1.20)	(0.01;0.30)	0.036*
HDL (mmol/L)	1.35 (1.11–1.52)	1.33 (1.14–1.54)	(−0.10;0.12)	0.88
FFA (mmol/L)	0.53 (0.42–0.69)	0.49 (0.35–0.63)	(−0.05;0.11)	0.36
ApoA1 (g/L)	1.35 (1.23–1.53)	1.36 (1.25–1.49)	(−0.06;0.07)	0.65
ApoB (g/L)	0.85 (0.71–1.06)	0.80 (0.68–0.94)	(−0.03;0.16)	0.17
LDL (mmol/L)	2.84 (2.28–3.57)	2.83 (2.25–3.20)	(−0.26;0.40)	0.84
Leptin (ng/mL)	8.10 (4.23–16.15)	7.69 (2.76–13.79)	(−2.61;4.33)	0.66
Adiponectin (µg/mL)	2.75 (1.01–4.29)	2.74 (1.88–4.16)	(−1.02;7.49)	0.91
Lpa (g/L)	0.13 (0.05–0.37)	0.11 (0.06–0.30)	(−0.03;0.06)	0.45
Glucose metabolism^d^				
Glucose (mmol/L)	5.0 (4.6–8.7)	4.9 (4.7–5.2)	(−0.1;0.3)	0.42
Insulin (pmol/L)	21.5 (13.0–55.0)	25.0 (13.0–34.0)	(−12.0;13.0)	0.86
HOMA	0.4 (0.4–0.8)	0.5 (0.4–0.6)	(−0.1; 0.2)	0.41
Other MetS-associated biomarkers				
Cystatin C (mg/L)	0.86 (0.81–0.94)	0.80 (0.74–0.86)	(0.03;0.11)	0.002**
Uric acid (mmol/L)	0.31 (0.25–0.40)	0.31 (0.26–0.34)	(−0.02; 0.05)	0.35
Urea (mmol/L)	5.3 (4.6–6.5)	5.1 (4.3–5.8)	(−0.2;0.8)	0.21
Creatinine (mg/mmol)	74 (67–85)	69 (63–78)	(1;10)	0.014*
hsCRP (mg/L)	1.42 (0.55–3.47)	1.27 (0.52–3.57)	(−0.62;0.97)	0.46
Vascular parameters^e^				
Central SBP (mmHg)	115 (105–126)	110 (101–122)	(−4;14)	0.28
Central DBP (mmHg)	76 (71–84)	77 (70–85)	(−5;4)	0.68
Central PP (mmHg)	38 (31–45)	33 (28–44)	(−1;10)	0.068
PP (mmHg)	46 (40–51)	46 (42–50)	(−4;2)	0.64
PPA	1.23 (1.04–1.44)	1.33 (1.07–1.69)	(−0.26;0.09)	0.40
Diameter CCA (mm)	6.38 (5.92–6.83)	6.40 (5.82–6.68)	(−0.25;0.33)	0.83
CIMT (µm)	523 (477–581)	531 (481–586)	(−40;21)	0.44
DC	25.6 (18.4–32.3)	28.3 (19.6–37.6)	(−7.2;3.7)	0.27
PWV (m/s)	6.9 (6.0–8.0)	7.0 (6.3–7.8)	(−0.7;0.4)	0.39

Significance codes: 0 *** 0.001 ** 0.01 * 0.05.

^a^Presented as median (IQR); ^b^Bootstrapped difference in medians; ^c^Subjects using lipid-lowering medication excluded (*n* = 4 survivors); ^d^Subjects with diabetes excluded (*n* = 6 survivors); ^e^Subjects using antihypertensive medication excluded (*n* = 6 survivors, *n* = 2 controls).

CCA, common carotid artery; CIMT, carotid intima media thickness; DC, distensibility coefficient; PP, pulse pressure; PPA, pulse pressure amplification; PWV, pulse wave velocity; S/DBP, systolic/diastolic blood pressure.

All vascular measurements were similar between survivors and controls.

### Influence of abdominal radiotherapy and nephrectomy on classic MetS components, biomarkers and vascular ultrasound measurements

Using univariable logistic regression, abdominal radiotherapy was associated with occurrence of MetS (odds ratio (OR) = 6.04, 95% CI = 2.04–17.89, *P* = 0.001), presence of two or more MetS components (OR = 3.36, 95% CI = 1.59–7.07, *P* = 0.001), as well as with all separate components of MetS (Table 4). Using multivariable regression analysis, adjusting for age, sex, smoking and socio-economic status, abdominal radiotherapy remained an independent risk factor for MetS occurrence (OR = 15.3, 95% CI = 3.21–73.36, *P* < 0.001), occurrence of two or more MetS components (OR = 3.23, 95% CI = 1.35–7.73, *P* = 0.009) as well as the MetS components high triglycerides, low HDL cholesterol and hypertension. Nephrectomy was not a risk factor for MetS occurrence (OR = 2.97, 95% CI = 0.98–8.97, *P* = 0.054). Using multivariable regression, nephrectomy was a risk factor for having two or more MetS components (OR = 2.78, 95% CI = 1.26–6.17, *P* = 0.012), high triglycerides or treatment (OR = 4.68, 95% CI = 1.66–13.19, *P* = 0.004) as well as hypertension or treatment (OR = 4.82, 95% CI = 2.05–11.29, *P* < 0.001).

**Table 4:** Uni- and multivariable regression of the effect of abdominal radiotherapy and nephrectomy on MetS and components.

	Univariable analysis			Multivariable analysis^a^		
	OR (s.e.)	95% CI	*P*-value	OR (s.e.)	95% CI	*P*-value
**The influence of abdominal radiotherapy**						
MetS	6.04 (0.554)	2.04;17.89	0.001**	15.3 (0.799)	3.21;73.36	<0.001***
≥2 MetS components	3.36 (0.380)	1.59;7.07	0.001**	3.23 (0.445)	1.35;7.73	0.009**
Abdominal obesity	<0.0001 (1659)	–	0.99	–	–	–
High triglycerides or treatment	5.70 (0.430)	2.45;13.24	<0.001***	7.01 (0.548)	2.39;20.52	<0.001***
Low HDL cholesterol or treatment	2.39 (0.389)	1.11;5.12	0.025*	2.94 (0.447)	1.23;7.07	0.016*
High blood pressure or treatment	4.24 (0.387)	1.99;9.06	<0.001***	5.11 (0.478)	2.00;13.02	<0.001***
High glucose or treatment	2.38 (0.431)	1.02;5.53	0.044*	2.53 (0.514)	0.92;6.93	0.071
**The influence of nephrectomy**						
MetS	2.97 (0.564)	0.98;8.97	0.054	–	–	–
≥2 MetS components	2.15 (0.354)	1.07;4.29	0.031*	2.78 (0.406)	1.26;6.17	0.012*
Abdominal obesity	0.41 (0.607)	0.13;1.35	0.14	–		–
High triglycerides or treatment	2.96 (0.426)	1.29;6.82	0.011*	4.68 (0.528)	1.66;13.19	0.004**
Low HDL cholesterol or treatment	1.22 (0.361)	0.60;2.47	0.59	–	–	–
High blood pressure or treatment	3.95 (0.375)	1.89;8.25	<0.001***	4.82 (0.435)	2.05;11.29	<0.001***
High glucose or treatment	1.55 (0.413)	0.69;3.48	0.29	–		–

Significance codes: 0 *** 0.001 ** 0.01 * 0.05.

^a^Corrected for age, sex, smoking and socio-economic status.

With regard to the biomarkers, abdominally irradiated subjects had higher triglycerides, FFA, apoB, LDL, cystatin C and urea levels (Supplementary Table 2A). Using multivariable linear regression analysis, abdominal radiotherapy remained an independent risk factor for higher triglycerides (β = 0.57, *P* = 0.002), higher FFA (β = 0.15, *P* = 0.008) and higher cystatin C (β = 0.08, *P* = 0.039) (Supplementary Table 2B). Nephrectomy was associated with higher cystatin C, uric acid, urea and creatinine levels (Supplementary Table 2C). Cystatin C (β = 0.12, *P* < 0.001), uric acid (β = 0.05, *P* = 0.006) and creatinine (β = 6.95, *P* = 0.042) remained significantly associated with nephrectomy in multivariable analysis (Supplementary Table 2D).

Ultrasonography revealed that abdominally irradiated survivors had significantly higher central systolic and diastolic blood pressure and PWV and lower DC (Supplementary Table 2A). In multivariable analysis, the association between abdominal radiation and higher central diastolic blood pressure remained significant (β = 5.39, *P* = 0.023) (Supplementary Table 2B). After nephrectomy, survivors had higher central systolic blood pressure, but this association was not significant in linear regression analysis (Supplementary Tables 2C and D). As peripheral blood pressure was higher as well, there was no clear added value of the vascular ultrasound measurements.

### Principal component analysis of biomarkers

Principal component analysis of the panel of 17 serum biomarkers in survivors yielded principal component 1 with explained variance of 24.2% and a pattern of ‘favourable lipids’ (high negative loading for HDL and adiponectin) towards ‘unfavourable lipids’ (high positive loading of triglycerides, apoB and LDL, as well as uric acid). Principal component 2 (PC2, orthogonal on PC1) explained 14.3% variance, with high negative loading reflected by ‘impaired glucose metabolism’ (HOMA and glucose (as well as leptin)) and high positive loading reflected by ‘kidney disease’ (creatinine and cystatin C (as well as HDL)). As all vectors in the biplot originate from the centre, high positive and negative loading on PC2 are two separate entities, that is, kidney disease is not associated with favourable glucose metabolism and neither is impaired glucose metabolism with good kidney function. This is only applicable to PC1 since one pattern – favourable to unfavourable lipids – can be distinguished along the whole axis.

**Figure 1:**
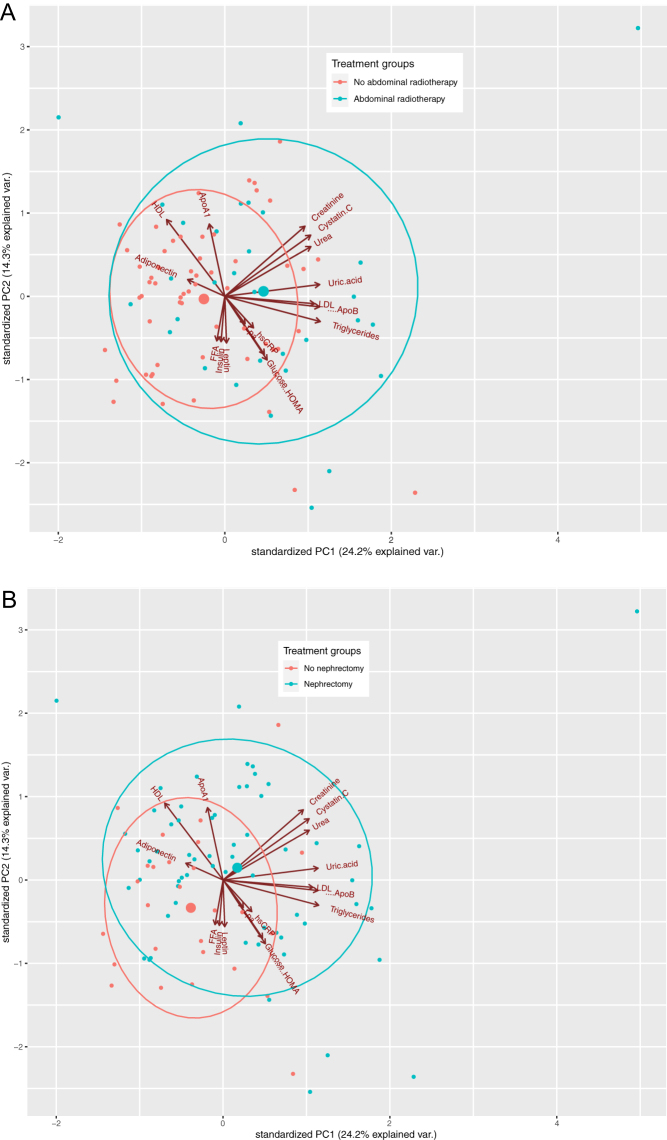
Biplots of PCA (principal components 1 and 2) of serum biomarkers, with the effect of abdominal radiotherapy (A) and nephrectomy (B). Score on PC1 and PC2 is a Z-score, based on loadings and Z-scores of biomarkers. ApoA1, apolipoprotein-A1; ApoB, apolipoprotein-B; FFA, free fatty acids; HDL, high density lipoprotein cholesterol; HOMA, homeostasis model assessment; LDL, low density lipoprotein cholesterol; Lpa, lipoprotein(a).

The effect of abdominal radiotherapy and nephrectomy on the two principal components is depicted in Fig. 1. Survivors who received abdominal radiotherapy had a higher positive loading on PC1, constituting the unfavourable profile (β = 1.45, *P* = 0.001). There was no significant influence of abdominal radiation on PC2 (β = 0.14, *P* = 0.68). Nephrectomy was associated with both higher positive loading on PC1 (unfavourable lipids, β = 1.13, *P* = 0.015) and PC2 (kidney disease, β = 0.75, *P* = 0.037).

## Discussion

This is the first report that describes the value of an integrated biomarker profile, defined by principal component analysis, and vascular ultrasound measurements, to estimate metabolic syndrome in long-term survivors of childhood nephroblastoma and neuroblastoma, in addition to classic parameters. We show that survivors more frequently develop MetS and that they have an unfavourable constitution of biomarkers in principal component 1 (PC1), particularly after abdominal radiotherapy. By using a principal component analysis, we could explore new variables better, since this analysis identifies individual contribution with no bias of multiple testing. This would not have been possible with another analysis, such as multiple correlation or regression. This unfavourable constitution of biomarkers consisted of a cluster of low HDL cholesterol and adiponectin and high triglycerides, LDL cholesterol, apoB and uric acid.

Low HDL and high triglycerides are already classic components of MetS in the NCEP-ATPIII classification; the other biomarkers have been reported as risk predictors for MetS and cardiovascular disease in the general population as well as in CCS (16, 17, 24, 25, 26). Therefore, we propose the addition of adiponectin, LDL, apoB and uric acid in a surveillance setting, particularly in abdominally irradiated survivors, to classify MetS as alternative for waist circumference.

Our suggestion to add biomarkers to the classical components of MetS is in line with the recently updated dyslipidaemia management guideline from the European Society of Cardiology (ESC) and the European Atherosclerosis Society (EAS), in which apoB analysis is recommended for cardiovascular risk assessment, particularly in people with high triglycerides, obesity, MetS and diabetes (27). apoB has been reported as a more sensitive marker of atherogenicity of cholesterol particles, in particular in insulin resistant patients. In those subjects, a relative abundance of dense, more atherogenic LDL particles can be present, which would be reflected by higher apoB levels (28). Uric acid is linked to metabolic syndrome in several ways: hyperuricemia contributes to the development of hypertension, insulin resistance and obesity (24). The observed inverse relation between adiponectin and abdominal radiotherapy is of interest, as it may suggest that local radiation damage leads to decreased endocrine function of the adipose tissue or a lower number of fat cells.

Our finding that abdominal radiotherapy is strongly associated with the development of MetS components in CCS is consistent with other studies and, more specifically, caused by radiation damage to the pancreas (29, 30, 31, 32). Additionally, abdominal radiotherapy can lead to underdevelopment of belly fat and musculature and to scoliosis. Hence, measurement of waist circumference underestimates adiposity. Previously, we reported that body composition is more accurately measured in these CCS by DXA-scan (3). The proposed use of additional biomarkers has the added advantage that this may be a cheaper and less burdensome diagnostic tool.

We found a moderate correlation between PC1 score and waist circumference in non-abdominally irradiated survivors (Pearson’s *r* = 0.64, substantially higher than the correlations of the separate biomarkers) (Supplementary Fig. 1), which supports the feasibility of this screening strategy. As a next step, to prove accuracy, sensitivity and cost-effectiveness of this strategy, replication in larger and independent cohorts is needed. Ultimately, for determining how the PC1 biomarkers could be incorporated in MetS classification, longitudinally collected information on solid endpoints (diabetes mellitus, cardio- and cerebrovascular morbidity and mortality) is needed.

Another finding was that principal component 2 was not of added value in determining MetS in abdominally irradiated survivors. By definition, PC2 explains less of the variance. Furthermore, there was no single discriminative pattern reflecting PC2 score and no difference in PC2 constitution was observed between abdominally irradiated and non-irradiated survivors.

Unexpectedly, the vascular ultrasound measurements were neither of evident added value in estimating MetS. We did observe some alterations suggestive of central arterial stiffness after abdominal radiotherapy: elevated central blood pressure and pulse wave velocity (PWV), and lower distensibility coefficient (DC); but after adjustment for potential covariates, only central blood pressure remained significantly associated with abdominal radiotherapy. Although central blood pressure is thought to better reflect cardiovascular risk as this represents the blood pressure in the coronary and cerebral arteries (33), we do not estimate this measurement of substantially added value, with peripherally measured blood pressure already being a classic MetS component. Although vascular abnormalities as observed by ultrasound can be early signs of MetS and its consequences, there can be variation in the development of these consequences, and the type of ultrasound patterns can vary as well. Therefore, in the aforementioned ESC/EAS dyslipidaemia guideline, it is postulated that assessment of arterial plaque burden can be considered as a risk modifier in individuals at low or moderate cardiovascular risk, in addition to standard cardiovascular risk assessment (27). We think that it is conceivable that this variation, as well as the relatively young age of our study cohort, may contribute to this unexpected finding. It could be that these vascular ultrasound measurements will be useful at an older age for early detection of atherosclerosis, so it would be useful to have longitudinal data. The question remains whether asymptomatic atherosclerosis detection would have implications compared to interventions for the other MetS components.

In the separate analysis of the biomarkers, we observed elevated cystatine C in abdominally radiated survivors, even without elevation of creatinine. This discrepant finding may be due to underdeveloped abdominal musculature and, if so, suggests that cystatine C is a more sensitive marker for assessing renal function in abdominally irradiated survivors (34).

The occurrence of MetS in our control group (3%) was relatively low, as MetS prevalence in The Netherlands at age 30–39 years has been reported as 10–20% (35). We confirmed the representativeness of our controls by comparing their metabolic profile with other published, similar aged, Dutch reference cohorts (36, 37).

Some limitations of the current study merit consideration. This was a cross-sectional study, providing information at one time point only. As the study population was still relatively young, it is anticipated that the prevalence of MetS will increase when the survivors age. The advantage of diagnosis at younger age is the opportunity to intervene timely, to prevent diabetes and cardiovascular disease. This is particularly beneficial in childhood cancer survivors who received other, direct cardiotoxic treatment, such as anthracyclines and radiotherapy. Furthermore, we did not have information about daily calorie intake and family history of metabolic syndrome, diabetes and cardiovascular disease in our study population. However, we did take siblings (60% of the control group) and partners as control and took the assumption that the calorie intake would be rather similar since they have a similar background. Family history is also most often similar between survivors and sibling controls.

Future research may focus on the validation of the use of adiponectin, LDL, apoB and uric acid in larger, independent cohorts of survivors, with longitudinal follow-up. It may also be of interest to study ratios of biomarkers that provide additional diagnostic accuracy of MetS in the general population, such as triglycerides/HDL-ratio and apoB-/apo-A1-ratio.

In conclusion, (young-)adult long-term survivors of childhood nephroblastoma and neuroblastoma, in particular after abdominal radiotherapy, frequently have MetS, defined by classic components, but also a novel, unfavourable integrated metabolic biomarker profile. This is important as the standard measurement of waist circumference after abdominal radiation is often infeasible in adult CCS. Our findings suggest that integrating the additional biomarkers identified in PCA – adiponectin, LDL, apoB and uric acid – may be useful to assess MetS, particularly in abdominally irradiated survivors. In contrast, vascular ultrasound measurements do not seem to be of additional value in estimating MetS at this relatively young age. Validation of our proposed screening strategy will be of importance to elucidate the higher risk of MetS, diabetes mellitus and cardiovascular disease in CCS, after previous intensive cancer treatment, which is still relatively disguised at young age, and to identify subgroups at greater risk at an early stage.

## Supplementary Material

Supplemental Table 1. Baseline characteristics of nephro- and neuroblastoma survivors separated.

Supplemental Table 2a. The effect of abdominal radiotherapy on biomarkers and vascular parameters.

Supplemental Table 2b. Uni- and multivariable bootstrap linear regression analysis of the influence of abdominal radiotherapy on biomarkers and vascular parameters.

Supplemental Table 2c. The effect of nephrectomy on biomarkers and vascular parameters.

Supplemental Table 2d. Uni- and multivariable bootstrap linear regression analysis of the influence of nephrectomy on biomarkers and vascular parameters.

Supplemental Figure 1. Correlation between waist and PC1 score in non-abdominally irradiated survivors.

## Declaration of interest

The authors declare that there is no conflict of interest that could be perceived as prejudicing the impartiality of the research reported.

## Funding

KiKa 2009-030, KOCR Foundation and Princess Máxima Centre core funding.
