# Supplementary material for: Metabolic syndrome detection with biomarkers in childhood cancer survivors
Source: Endocr Connect. 2020 Jun 18;9(7):676–86. doi: 10.1530/EC-20-0144 (PMC7424353; doi:10.1530/EC-20-0144)
Supplement: Supplemental Table 1. Baseline characteristics of nephro- and neuroblastoma survivors separated. [file supplementary_table_1.pdf]

Supplemental Table 1. Baseline characteristics of nephro- and neuroblastoma survivors separated.

	**Nephroblastoma**	**Neuroblastoma**	**Bootstrap 95% CI**	**P-value**
Number	67	36		
Male sex	39 (58.2%)	15 (41.7%)	n.a.	0.16[1]
Age at follow-up (years) #	30.2 [25.2 – 39.4]	29.6 [25.1 – 33.4]	[-4.0;5.9]	0.85[2]
Age at diagnosis (years) #	3.3 [1.8 – 5.2]	0.8 [0.3 – 1.9]	[1.4;3.8]	<0.001[2]***
Follow-up time^ (years) #	26.3 [19.3 – 32.7]	27.8 [21.7 – 30.9]	[-6.3;3.1]	0.54[2]
BMI (kg/m^2) #	24.6 [21.7 – 27.1]	24.1 [21.1 – 26.0]	[-1.7;2.6]	0.72[2]
Systolic BP (mmHg) #	123 [118 – 133]	124 [115 – 129]	[-6;6]	0.62[2]
Diastolic BP (mmHg) #	77 [73 – 83]	75 [72 – 84]	[-4;5]	0.20[2]
Medication use				
Lipid-lowering	3 (4.5%)	1 (2.8%)	n.a.	1[3]
Diabetes	5 (7.5%)	1 (2.8%)	n.a.	0.66[3]
Antihypertensive	5 (7.5%)	1 (2.8%)	n.a.	0.66[3]
Physical activity score #	8140 [6634 – 12070]	6685 [5805 – 8423]	[-248;2560]	0.10[2]
Smoking			n.a.	0.29[1]
Non-smoker	44 (65.7%)	18 (50.0%)		
Former smoker	8 (11.9%)	7 (19.4%)		
Smoker	15 (22.4%)	11 (30.6%)		
Socio-economic status			n.a.	1[3]
Low	14 (20.9%)	8 (22.2%)		
Medium	24 (35.8%)	12 (33.3%)		
High	29 (43.3%)	16 (44.4%)		
Nephrectomy	67 (100%)	7 (19.4%)	n.a.	<0.001[1]***
Adrenalectomy	33 (49.3%)	14 (38.9%)	n.a.	0.42[1]
Abdominal radiotherapy	35 (52.2%)	7 (19.4%)	n.a.	0.003[1]**
Pancreas	24 (35.8%)	7 (19.4%)		0.23[1]
Flank	17 (26.6%)	0		0.002[1]**
Cumulative dose radiotherapy (Gy) #	21 [20 – 30]	20 [19.6 – 20.5]	[-0.7;8.6]	0.064[2]
Chemotherapy	59 (88.1%)	31 (86.1%)	n.a.	0.76[3]
Vincristine	50 (74.6%)	15 (41.7%)		0.002[1]**
Actinomycine	48 (71.6%)	0		<0.001[1]***
Anthracyclines	18 (26.9%)	12 (33.3%)		0.64[1]
Cyclofosfamide	2 (3.0%)	29 (80.6%)		<0.001[1]***
Cisplatin	0	7 (19.4%)		<0.001[3]
Teniposide	0	6 (16.7%)		0.001[3]
Dacarbazine	2 (3.0%)	0		0.54[3]
Ifosfamide	2 (3.0%)	0		0.54[3]
Corticosteroids	0	2 (5.6%)	n.a.	0.12[3]

BP = blood pressure; n.a. = not applicable.
# Presented as median [IQR]; ^ Time after cessation of treatment.
Significance codes: 0 *** 0.001 ** 0.01 * 0.05
[1] Chi-squared test, [2] Bootstrapped difference in medians, [3] Fisher's exact test
