# Supplementary material for: Metabolic syndrome detection with biomarkers in childhood cancer survivors
Source: Endocr Connect. 2020 Jun 18;9(7):676–86. doi: 10.1530/EC-20-0144 (PMC7424353; doi:10.1530/EC-20-0144)
Supplement: Supplemental Table 2a. The effect of abdominal radiotherapy on biomarkers and vascular parameters. [file supplementary_table_2.pdf]

Supplemental Table 2a. The effect of abdominal radiotherapy on biomarkers and vascular parameters.

Variable	Abdominal radiotherapy (n=42) #	No abdominal radiotherapy (n=61) #	95% CI ^	P-value ^
Biomarkers				
Lipid metabolism[1]				
Triglycerides (mmol/L)	1.35 [0.81 – 2.09]	0.91 [0.70 – 1.08]	[0.06;0.88]	0.024*
HDL (mmol/L)	1.32 [1.08 – 1.52]	1.35 [1.12 – 1.52]	[-0.19;0.13]	0.74
FFA (mmol/L)	0.57 [0.44 – 0.74]	0.50 [0.38 – 0.64]	[0.01;0.17]	0.026*
ApoA1 (g/L)	1.35 [1.21 – 1.58]	1.35 [1.25 – 1.49]	[-0.10;0.16]	0.75
ApoB (g/L)	0.99 [0.81 – 1.18]	0.81 [0.65 – 0.97]	[0.07;0.30]	0.004**
LDL (mmol/L)	3.19 [2.71 – 3.79]	2.55 [2.15 – 3.18]	[0.15;1.09]	0.004**
Leptin (ng/mL)	9.00 [4.98 – 18.38]	7.60 [3.67 – 12.90]	[-2.95;6.81]	0.51
Adiponectin (μg/mL)	2.30 [0.56 – 4.31]	2.83 [1.53 – 4.15]	[-2.09;1.22]	0.64
Lpa (g/L)	0.09 [0.03 – 0.45]	0.13 [0.05 – 0.35]	[-0.10;0.17]	0.65
Glucose metabolism[2]				
Glucose (mmol/L)	5.0 [4.8 – 5.5]	4.9 [4.6 – 5.3]	[-0.2;0.4]	0.36
Insulin (pmol/L)	18.0 [13.0 – 44.0]	24.0 [13.0 – 58.0]	[-22.0;13.0]	0.48
HOMA	0.4 [0.4 – 0.8]	0.4 [0.4 – 0.8]	[-0.3; 0.3]	0.46
Other MetS-associated biomarkers				
Cystatin C (mg/L)	0.88 [0.83 – 0.97]	0.85 [0.77 – 0.90]	[0.00;0.09]	0.048*
Uric acid (mmol/L)	0.35 [0.29 – 0.44]	0.30 [0.24 – 0.38]	[-0.01;0.10]	0.094
Urea (mmol/L)	5.7 [5.1 – 6.8]	5.0 [4.5 – 5.9]	[0.1;1.5]	0.006**
Creatinine (mg/mmol)	75 [68 – 84]	74 [66 – 85]	[-7;9]	0.83
hsCRP (mg/L)	1.62 [0.77 – 3.35]	1.42 [0.39 – 3.79]	[-0.90;1.61]	0.65
Vascular parameters[3]				
Central SBP (mmHg)	124 [114 – 132]	109 [101 – 122]	[8;20]	<0.001***
Central DBP (mmHg)	84 [76 – 80]	74 [69 – 77]	[4;15]	<0.001***
Central PP (mmHg)	40 [34 – 47]	37 [30 – 44]	[-2;9]	0.19
PP (mmHg)	45 [41 – 53]	46 [40 – 49]	[-4;6]	0.95
PPA	1.23 [1.06 – 1.40]	1.30 [1.03 – 1.48]	[-0.21;0.15]	0.52
Diameter CCA (mm)	6.46 [5.93 – 6.90]	6.32 [5.92 – 6.83]	[-0.30;0.53]	0.47
CIMT (μm)	552 [482 – 595]	509 [458 – 569]	[-2;71]	0.052
DC	20.6 [15.7 – 26.1]	30.3 [21.3 – 39.8]	[-13.9;3.1]	0.004**
PWV (m/s)	8.0 [6.1 – 8.8]	6.6 [6.0 – 7.3]	[0.2;1.9]	0.016*

S/DBP = systolic/diastolic blood pressure; PP = pulse pressure; PPA = pulse pressure amplification; CCA = common carotid artery; CIMT = carotid intima media thickness; DC = distensibility coefficient; PWV = pulse wave velocity.

Presented as median [IQR]; ^ Bootstrapped difference in medians

Significance codes: 0 *** 0.001 ** 0.01 * 0.05

[1] Subjects using lipid-lowering medication excluded (n=3 abdominal radiotherapy, n=1 no abdominal radiotherapy)

[2] Subjects with diabetes excluded (n=5 abdominal radiotherapy, n=1 no abdominal radiotherapy)

[3] Subjects using antihypertensive medication excluded (n=4 abdominal radiotherapy, n=2 no abdominal radiotherapy)
