# Supplementary material for: Metabolic syndrome detection with biomarkers in childhood cancer survivors
Source: Endocr Connect. 2020 Jun 18;9(7):676–86. doi: 10.1530/EC-20-0144 (PMC7424353; doi:10.1530/EC-20-0144)
Supplement: Supplemental Table 2b. Uni- and multivariable bootstrap linear regression analysis of the influence of abdominal radiotherapy on biomarkers and vascular parameters. [file supplementary_table_3.pdf]

Supplemental Table 2b. Uni- and multivariable bootstrap linear regression analysis of the influence of abdominal radiotherapy on biomarkers and vascular parameters.

		Univariable analysis		Multivariable analysis[1]	
**Variable**	**P-value bootstrap difference medians**	**Beta (s.e.)**	**P-value**	**Beta (s.e.)**	**P-value**
Triglycerides	0.024	0.614 (0.165)	<0.001***	0.572 (0.154)	0.002**
FFA	0.026	0.123 (0.049)	0.008**	0.151 (0.059)	0.008**
ApoB	0.004	0.168 (0.054)	0.002**	0.083 (0.068)	0.19
LDL	0.004	0.469 (0.170)	0.008**	0.043 (0.235)	0.83
Cystatin C	0.048	0.065 (0.032)	0.041*	0.076 (0.039)	0.039*
Urea	0.006	0.895 (0.373)	0.011*	0.690 (0.465)	0.10
Central SBP	<0.001	10.226 (3.687)	0.008**	6.029 (5.033)	0.19
Central DBP	<0.001	9.122 (2.024)	<0.001***	5.385 (2.929)	0.023*
DC	0.004	-8.578 (2.810)	0.004**	-2.672 (3.279)	0.44
PWV	0.016	0.935 (0.451)	0.025*	0.294 (0.456)	0.53

Significance codes: 0 *** 0.001 ** 0.01 * 0.05

[1] Corrected for age, sex, smoking and socio-economic status.
