# Supplementary material for: Metabolic syndrome detection with biomarkers in childhood cancer survivors
Source: Endocr Connect. 2020 Jun 18;9(7):676–86. doi: 10.1530/EC-20-0144 (PMC7424353; doi:10.1530/EC-20-0144)
Supplement: Supplemental Table 2c. The effect of nephrectomy on biomarkers and vascular parameters. [file supplementary_table_4.pdf]

Supplemental Table 2c. The effect of nephrectomy on biomarkers and vascular parameters.

Variable	Nephrectomy (n=74) #	No nephrectomy (n=29) #	95% CI ^	P-value ^
Biomarkers				
Lipid metabolism[1]				
Triglycerides (mmol/L)	0.98 [0.77 – 1.58]	0.90 [0.70 – 1.19]	[-0.16;0.37]	0.43
HDL (mmol/L)	1.35 [1.09 – 1.55]	1.32 [1.16 – 1.48]	[-0.13;0.16]	0.72
FFA (mmol/L)	0.50 [0.40 – 0.61]	0.65 [0.49 – 0.77]	[-0.26;0.01]	0.054
ApoA1 (g/L)	1.38 [1.24 – 1.54]	1.31 [1.20 – 1.51]	[-0.04;0.16]	0.18
ApoB (g/L)	0.85 [0.70 – 1.05]	0.86 [0.74 – 1.08]	[-0.17;0.13]	0.79
LDL (mmol/L)	2.83 [2.20 – 3.54]	2.92 [2.45 – 3.63]	[-0.69;0.28]	0.43
Leptin (ng/mL)	8.37 [3.64 – 18.38]	8.10 [5.44 – 12.90]	[-5.47;3.66]	0.86
Adiponectin (µg/mL)	2.47 [0.98 – 4.31]	3.02 [1.81 – 4.13]	[-1.58;0.55]	0.26
Lpa (g/L)	0.13 [0.05 – 0.35]	0.11 [0.05 – 0.39]	[-0.11;0.08]	0.80
Glucose metabolism[2]				
Glucose (mmol/L)	5.0 [4.7 – 5.3]	4.8 [4.5 – 5.4]	[-0.1;0.5]	0.086
Insulin (pmol/L)	29.0 [13.0 – 55.0]	13.0 [13.0 – 49.0]	[-6.0;23.0]	0.088
HOMA	0.6 [0.4 – 0.9]	0.4 [0.4 – 0.8]	[-0.1; 0.3]	0.060
Other MetS-associated biomarkers				
Cystatin C (mg/L)	0.88 [0.84 – 0.99]	0.81 [0.72 – 0.86]	[0.03;0.16]	0.002**
Uric acid (mmol/L)	0.35 [0.27 – 0.43]	0.26 [0.21 – 0.31]	[0.02;0.13]	0.002**
Urea (mmol/L)	5.4 [4.8 – 6.6]	5.0 [4.2 – 5.7]	[-0.2;1.0]	0.084
Creatinine (mg/mmol)	79 [69 – 89]	69 [63 – 75]	[2;16]	0.004**
hsCRP (mg/L)	1.62 [0.72 – 3.48]	1.42 [0.39 – 3.09]	[-0.93;1.45]	0.46
Vascular parameters[3]				
Central SBP (mmHg)	118 [108 – 127]	108 [100 – 123]	[0;19]	0.050*
Central DBP (mmHg)	77 [72 – 85]	75 [71 – 79]	[-2;7]	0.17
Central PP (mmHg)	39 [34 – 47]	34 [30 – 43]	[-3;10]	0.14
PP (mmHg)	46 [40 – 53]	45 [41 – 48]	[-3;5]	0.62
PPA	1.23 [0.99 – 1.41]	1.31 [1.13 – 1.54]	[-0.30;0.09]	0.43
Diameter CCA (mm)	6.30 [5.87 – 6.82]	6.57 [6.13 – 6.93]	[-0.72;0.21]	0.32
CIMT (µm)	521 [479 – 582]	523 [461 – 570]	[-43;53]	0.81
DC	24.0 [15.8 – 39.8]	29.9 [24.0 – 31.9]	[-10.7;1.5]	0.13
PWV (m/s)	6.8 [6.0 – 8.0]	6.9 [6.1 – 7.5]	[-0.7;0.7]	0.79

S/DBP = systolic/diastolic blood pressure; PP = pulse pressure; PPA = pulse pressure amplification; CCA = common carotid artery; CIMT = carotid intima media thickness; DC = distensibility coefficient; PWV = pulse wave velocity.

Presented as median [IQR]; ^ Bootstrapped difference in medians

Significance codes: 0 *** 0.001 ** 0.01 * 0.05

[1] *Subjects using lipid-lowering medication excluded (n=3 nephrectomy, n=1 no nephrectomy)*

[2] *Subjects with diabetes excluded (n=5 nephrectomy, n=1 no nephrectomy)*

[3] *Subjects using antihypertensive medication excluded (n=6 nephrectomy)*
