# Supplementary material for: Metabolic syndrome detection with biomarkers in childhood cancer survivors
Source: Endocr Connect. 2020 Jun 18;9(7):676–86. doi: 10.1530/EC-20-0144 (PMC7424353; doi:10.1530/EC-20-0144)
Supplement: Supplemental Table 2d. Uni- and multivariable bootstrap linear regression analysis of the influence of nephrectomy on biomarkers and vascular parameters. [file supplementary_table_5.pdf]

Supplemental Table 2d. Uni- and multivariable bootstrap linear regression analysis of the influence of nephrectomy on biomarkers and vascular parameters.

		**Univariable analysis**		**Multivariable analysis[1]**	
**Variable**	**P-value bootstrap difference medians**	**Beta (s.e.)**	**P-value**	**Beta (s.e.)**	**P-value**
Cystatin C	0.002	0.128 (0.027)	<0.001***	0.115 (0.027)	<0.001***
Uric acid	0.002	0.075 (0.017)	<0.001***	0.045 (0.013)	0.006**
Creatinine	0.004	12.062 (2.899)	0.002**	6.954 (2.640)	0.042*
Central SBP	0.050	8.233 (4.089)	0.054	-	-

Significance codes: 0 *** 0.001 ** 0.01 * 0.05

[1] Corrected for age, sex, smoking and socio-economic status
