# Supplementary figures and images for: Metabolic syndrome detection with biomarkers in childhood cancer survivors
Source: Endocr Connect. 2020 Jun 18;9(7):676–86. doi: 10.1530/EC-20-0144 (PMC7424353; doi:10.1530/EC-20-0144)

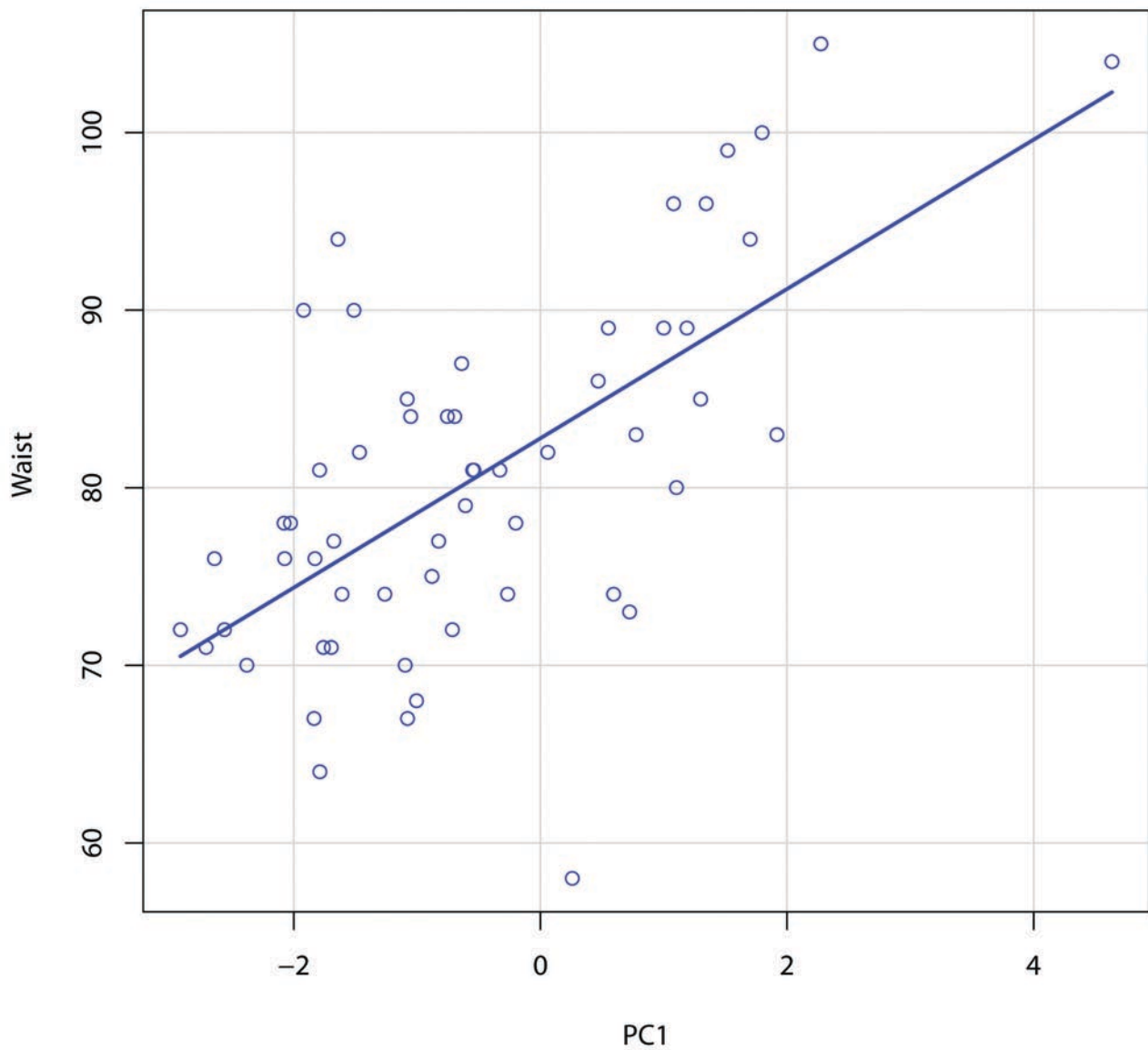

Supplement: Supplemental Figure 1. Correlation between waist and PC1 score in non-abdominally irradiated survivors. [file supplementary_figure_1.pdf]
